# Live video rate volumetric OCT imaging of the retina with multi-MHz A-scan rates

**DOI:** 10.1371/journal.pone.0213144

**Published:** 2019-03-28

**Authors:** Jan Philip Kolb, Wolfgang Draxinger, Julian Klee, Tom Pfeiffer, Matthias Eibl, Thomas Klein, Wolfgang Wieser, Robert Huber

**Affiliations:** 1 Institut für Biomedizinische Optik, Universität zu Lübeck, Lübeck, Germany; 2 Optores GmbH, München, Germany; University of Melbourne, AUSTRALIA

## Abstract

Surgical microscopes are vital tools for ophthalmic surgeons. The recent development of an integrated OCT system for the first time allows to look at tissue features below the surface. Hence, these systems can drastically improve the quality and reduce the risk of surgical interventions. However, current commercial OCT-enhanced ophthalmic surgical microscopes provide only one additional cross sectional view to the standard microscope image and feature a low update rate. To present volumetric data at a high update rate, much faster OCT systems than the ones applied in today’s surgical microscopes need to be developed. We demonstrate live volumetric retinal OCT imaging, which may provide a sufficiently large volume size (330x330x595 Voxel) and high update frequency (24.2 Hz) such that the surgeon may even purely rely on the OCT for certain surgical maneuvers. It represents a major technological step towards the possible application of OCT-only surgical microscopes in the future which would be much more compact thus enabling many additional minimal invasive applications. We show that multi-MHz A-scan rates are essential for such a device. Additionally, advanced phase-based OCT techniques require 3D OCT volumes to be detected with a stable optical phase. These techniques can provide additional functional information of the retina. Up to now, classical OCT was to slow for this, so our system can pave the way to holographic OCT with a traditional confocal flying spot approach. For the first time, we present point scanning volumetric OCT imaging of the posterior eye with up to 191.2 Hz volume rate. We show that this volume rate is high enough to enable a sufficiently stable optical phase to a level, where remaining phase errors can be corrected. Applying advanced post processing concepts for numerical refocusing or computational adaptive optics should be possible in future with such a system.

## 1. Introduction

Optical Coherence Tomography (OCT) [1] is a non-invasive imaging modality, which uses usually near-infrared light to create three dimensional images with μm-scale resolution. Typically, it features 1–2 mm penetration depth for scattering tissue. Also, it is perfectly suited for retinal imaging [2, 3], as the lens and the vitreous body of the human eye consist mostly of water, nearly transparent to some parts of the near-infrared spectrum [4]. Here, OCT’s unique capability of creating detailed cross-sectional views is very valuable for diagnosis of retinal diseases [5]. While ophthalmic imaging is still the main application for OCT, it also has found many other applications, for example intravascular imaging [6], dermatology [7] or even non-destructive testing of materials [8].

In most cases, the acquisition of a single volume takes several seconds, which is sufficient for most OCT use cases. Nevertheless, there are some applications, where it might be desirable to obtain volumes at video rates (4D-OCT, i.e. 3D over time).

The greatest drive in developing retinal *real time 4D-OCT* systems has been its application for surgical guidance. So far, only one implementation of densely sampled retinal real-time 4D-OCT has been published with volume sizes of 300×100 A-scans at low volume rates of 3.33 Hz [9]. There has also been a conference presentation, where 400×96 A-scans at 10.85 Hz were presented [10]. However, the spectrum was split [11] to gain the necessary A-scan rate at the cost of a reduced axial resolution. Most research is limited to two dimensional B-scans being displayed at video rate [12–14]. The limitation to a single cross-sectional view suffers from the problem that the region of interest, where a surgical maneuver should be performed, and the locations of the B-scans always have to be realigned. Instrument tracking [15] automates this step, but adds complexity to the system. Nevertheless, having a cross-sectional view in addition to a frontal view already simplifies existing maneuvers like deep anterior lamellar keratoplasty (DALK) [16], descemet membrane endothelial keratoplasty (DMEK) [17] or repairing a retinal detachment [18]. Besides, it also makes training for surgeons easier [19]. Therefore, this technique has already been commercialized and is available as an OCT-enhanced surgical microscope from Carl-Zeiss AG and Haag-Streit GmbH (Optmed, Lübeck, Germany) or as an additional module by Leica AG.

All solutions mentioned above have in common that the surgeon cannot rely on the OCT image alone, but still needs to share his attention between the OCT and the regular microscope view. A sufficiently high definition volumetric OCT image has the advantage of a much higher plasticity and better perception of structures over a standard stereoscopic view. Since OCT is a type of confocal scanning microscope, also the contrast of OCT intensity projections can be superior to standard reflection microscopes. So high definition 4D OCT has the potential to completely replace the wide field channel in surgical microscopes at some point in the future. However, OCT does not provide any color information, but this might easily be added by implementing a multi-laser scanning laser ophthalmoscope to map actual colors onto the OCT data [20, 21]. Of course thorough translational studies would have to be performed to determine the feasibility of OCT-only surgery from a medical point of view.

For an ophthalmic OCT-only microscope, we propose six requirements: (1) The A-scan density should correspond to a similar optical resolution of the regular microscope. Typical optical resolutions of ophthalmic surgical microscopes are on the order of 10 μm to 20 μm [22]. Over a field of view of 20°×20° or 6×6 mm^2^, 600×600 to 300×300 A-scans would be necessary to achieve the same sampling density. (2) The volumes should be updated at a rate that allows a fluent perception of motion. According to literature, this rate lies around 30 Hz [23]. (3) A high sensitivity of the system should ensure a reasonable image quality. We proposed a sensitivity close to 90 dB or higher in [24]. (4) The time lag between the action and the display should be below the human reaction time to provide instantaneous feedback to the user. It was suggested that this value is around 100–150 ms and below [25, 26]. (5) The wavelength of the system should penetrate the vitreous body without strong absorption. This restricts the system to the use of light in the visible and near-infrared spectrum with wavelengths below 900 nm or around 1060 nm [4]. (6) The imaging range of the system should be sufficient to display the entire retina and 1–2 mm anterior of the retinal surface to provide ample spatial context to the surgeon regarding the location of the instruments. Additionally, some retinal pathologies such as retinal detachments require a large axial imaging range for visualization. It was found that 3.7 mm should cover even most severe retinal detachments [9].

Considering requirements (1) and (2) we calculate the minimum A-scan rate to 300 × 300 × 30 Hz = 2.7 MHz, if we neglect dead time due to scanning. This shows that only systems with MHz speed are suitable. Only few systems are such fast while still sustaining a sufficient sensitivity according to (3): In SD-OCT, there is an approach with parallelizing four spectrometers reaching 1 MHz A-Scan rate [27]. Regarding SS-OCT, a MEMS-tunable-VECSEL source was demonstrated with 1 MHz [28] and the FDML laser with up to 5.2 MHz per spot at 1300 nm wavelength [29] and 3.35 MHz per spot a 1060 nm wavelength [24]. If parallelization approaches like line-field SS-OCT are included, 1 MHz is also feasible with a slower sweeping light source [30].

Additionally, for an ophthalmic OCT-only microscope, real time processing and display is essential as stated in (4). This adds complexity to the system as it makes advanced parallel processing necessary, which is preferably performed on a graphics processing unit (GPU) [31] or FPGA. In addition, timing and jitter become more critical. Although there have been some implementations [32–35], only Wieser et al. [34] combined it with suitably high A-scan rate to meet (1) and (2). Still, in this publication a 1300 nm FDML laser was used and therefore does not fulfill criteria (5)–it has no appropriate wavelength for imaging the retina. FDML lasers at 1060 nm are more difficult to construct and to handle due to the higher dispersion in optical fibers and their higher susceptibility to polarization mode dispersion. Additionally, the power exposure limits are significantly lower for the posterior eye than for other samples like skin. This results in a lower shot noise limited sensitivity, making it more difficult to obtain a sufficient image quality as stated in requirement (3). Therefore, the realization of a live 4D OCT at 1050 nm wavelength is much more challenging than at 1300 nm wavelength.

Besides an ophthalmic OCT microscope, we could also envision the application of such a system in optical coherence microscopy (OCM) [36]. Here similar criteria would apply–especially the wavelength should be as close to the visible spectrum as possible, as most microscopy optics are designed for this wavelength range and as shorter wavelengths provide a higher diffraction-limited transverse resolution. Besides this, it would also be possible to integrate computational optics for numerical refocusing into the live processing to extend the depth of field [37, 38].

*4D-OCT without real-time display* (4D-OCT with offline processing) also has some applications at the posterior eye, where a short update interval between the individual volumes is required. First, this is the case because dynamic processes are of interest. In non-ophthalmic applications it has been demonstrated, that one can visualize thermal damage to a strand of hair [39], show the dynamics of a beating frog embryo heart [40] or do OCT-based microangiography (OMAG) [41–43]. Second, 4D-OCT with offline processing can also be beneficial to phase-based applications where the phase within a volume or between consecutive volumes should change as little as possible. The requirement of a fast imaging system becomes more critical in this case, as one usually has more motion originating from multiple sources: Mechanical instabilities in the system, head motion of the patient and microsaccades if no anesthesia is applied. One example for a possible application is numerical refocusing or aberration correction to visualize individual photoreceptors or other small features without adaptive optics [44–46]. Here, the demand for high volume rates is less critical, as a stable phase is only required over one volume. It was shown that a B-scan rate of 1.5 kHz is sufficient [47]. However, to the best of our knowledge, this was only shown with full field OCT, line field OCT and an en face OCT so far, but not with a volumetric flying spot OCT. Flying spot OCT may offer advantages considering contrast and dynamic range. Another example is the visualization of pulsatile waves and measuring their velocity to determine the biomechanical properties of retinal blood vessels [48]. Moreover, it is possible to measure the axial expansion of photoreceptors in response to an optical stimulus [49]. The two last applications are more demanding, as they compare the phase between multiple volumes, where motion artifacts changing the phase should be avoided and the time interval between the volumes should be short enough to evaluate the dynamics of the process. Therefore, they have been exclusive to full field OCT because it intrinsically features a stable phase within a volume and is capable of high volume rates. However, full field OCT lacks a sufficient sensitivity and the absence of confocal gating to suppress multiply scattered photons which makes it impractical for choroidal imaging. Typical volume rates were on the order of 100–200 Hz. Obviously, it would be very interesting to implement these techniques in a flying spot OCT, but this requires a high-volume rate.

In this publication, we combine our fast MHz FDML laser at 1060nm [24, 50] to create high volume rate OCT images with and without our live processing demonstrated previously at 1300nm. We present live 4D OCT at 1060 nm with 330x330x595 voxels at 24.2 V/s corresponding to 1.58 GVoxels/s, which sets a new record for live OCT imaging and processing and enables new perspectives towards an OCT-only surgical microscope. Additionally, we take first steps towards phase stable imaging with a flying spot OCT by increasing the volume rate of 4D OCT imaging up to 191.2 V/s. We start with a detailed outline of our setup, then we show examples of retinal imaging and discuss the challenges related to these experiments such as eye motion, image processing and the necessity of multi-MHz A-scan rates.

## 2. Experimental setup

For the investigation of the *live 4D OCT* with focus on surgical guidance, we use a slightly different setup than for the *non-live OCT imaging* focusing on potential applications regarding phase stable imaging. An overview of the two configurations can be found in Table 1. In this chapter, we describe in detail our setups by starting with the FDML laser itself. Next we will present our interferometer and the scanning optics, then we will discuss our data acquisition and processing and finally we will describe our synchronization strategies for both setups.

**Table 1:** Imaging setups.

setup	non-live imaging	live imaging
possible applications	numerical refocusing and aberration corrections, blood vessel property assessment [48], measurement of photoreceptor stimulation [49]	surgical guidance, OCM
A-scan rate	1.67 MHz	3.34 MHz
photodetector	Wieserlabs WL-BPD1GA 1 GHz bandwidth	Thorlabs PDB480C-AC 1.6 GHz bandwidth (modified)^1^
digitizer card (ADC)	Alazartech ATS9360 1.8 GS/s (12 bit sampling depth, 800 MHz analog bandwidth)	Alazartech ATS9373 4 GS/s (12 bit sampling depth, 2 GHz analog bandwidth)
live processing solution	none	Optores OGOP software (running on Nvidia Geforce GTX 690 and GTX680)
imaging range^2^	4.2 mm	4.7 mm
axial resolution^2^	13 μm	
transverse resolution^3^	12.5 μm	
x-mirror scan speed	4.294 kHz resonant scanner	
scan protocol(s)	160x160 (A); 160x80 (B); 160x40 A-scans (C)	330x330 A-scans
field of view	15°x15°(A), 15°x7°(B), 15°x3°(C)	25°x25°
# of voxels along z-axis	535	595
volume rate	52.4 Hz (A); 102.2 Hz (B); 191.2 Hz (C)	24.2 Hz
voxel rate	0.6 GVoxel/s	1.58 GVoxel/s
sensitivity	90 dB	87 dB

^1^ The internal FC/APC connectors with SMF28 fiber were replaced with FC/APC connectors with Hi1060 fiber.

^2^ in air

^3^ FWHM of the spot size on the retina

### 2.1 The FDML laser

**Fig 1:**
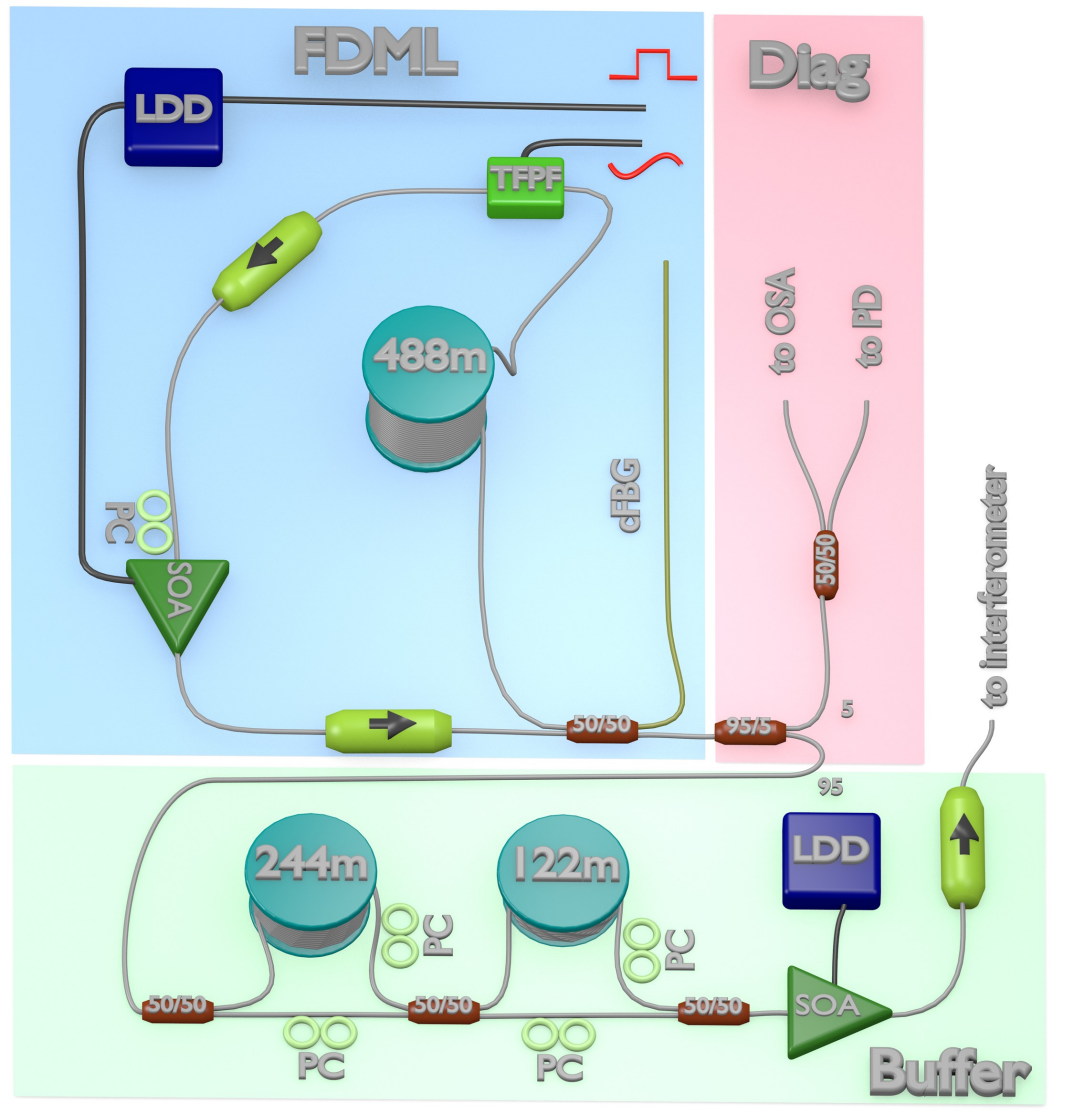
Schematic of the FDML laser with a 4x buffer stage (1.67 MHz) used in the experiments. The 3.34 MHz version has an additional fiber spool and coupler in the buffer stage. LDD: laser diode driver, TFPF: home build tunable Fabry-Pérot filter, PC: polarization controller, arrow: optical isolator, SOA: semiconductor optical amplifier, cFBG: chirped fiber Bragg grating, OSA: optical spectrum analyzer, PD: photodiode. Numbers on spools show fiber length. The LDD in the laser cavity receives a rectangular signal, the TFPF a sinusoidal. Buffer: buffer stage; Diag: laser diagnostics.

The schematic of the light source of our SS-OCT system is shown in Fig 1. It is an improved version of the FDML laser [51] presented in [24]. Due to advances in semiconductor optical amplifier (SOA) technology the SOA (Innolume SOA-1020-110-Hi1060-27dB for non-live imaging or SOA-1060-90-HI-30dB for live imaging) alone provides sufficient gain to operate the laser with at least 70 nm sweeping bandwidth around 1050 nm center wavelength and no additional intracavity Ytterbium amplifier is required. The chirped fiber Bragg grating pre-compensates the dispersion of the 488 m long fiber spool and the rest of the cavity to equalize the roundtrip time for every wavelength. In the following experiments we use two different configurations of the buffer stage [52] to achieve different A-scan rates: For *non-live OCT* with a 4x buffer stage, the SOA in the FDML cavity is modulated with a fast laser diode driver (LDD, Wieserlabs WL-LDC10D) with a 25% duty cycle. The attached buffer stage quadruples the fundamental FDML laser frequency of 417 kHz to 1.67 MHz. In case of *live OCT imaging* an 8x buffer stage in combination with a 12.5% modulation is used and the fundamental frequency is multiplied to 3.34 MHz. In both cases, the output of the bufferstage is amplified with a booster SOA of the same type as in the cavity before being guided to the interferometer.

### 2.2 The interferometer and scanning optics

**Fig 2:**
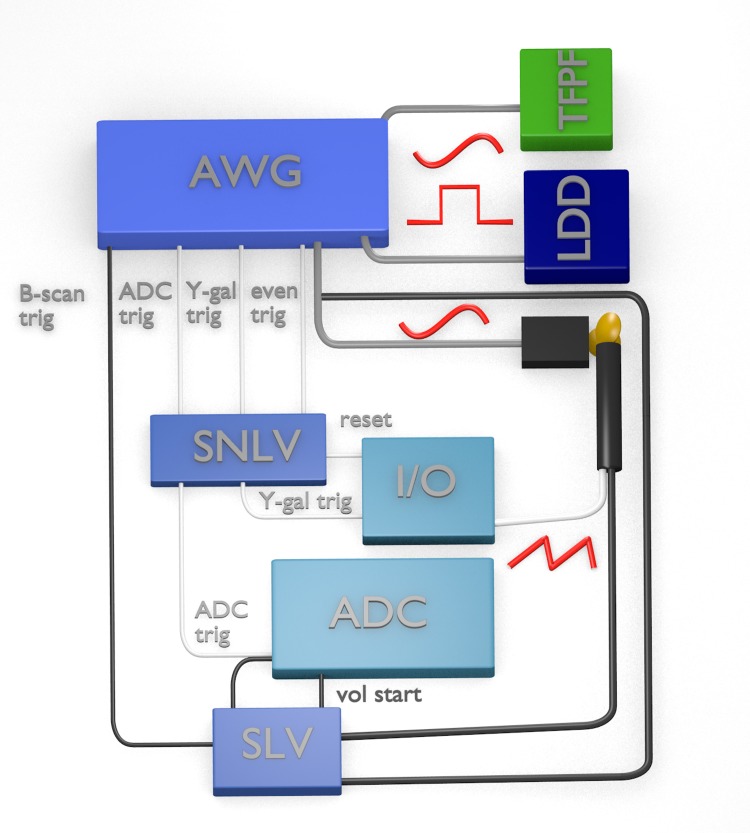
Interferometer design including sample arm. **DC: dispersion compensation.** BPD: balanced photodetector, recal: recalibration arm, reference: reference arm, PC: polarization controller, X-mirror: x-axis resonant scanner, Y-mirror: Y-axis galvanometer scanner, L1 and L2: lens group 1 and 2, DM: dichroic mirror, FT: fixation target (attenuated LED projector).

The basic layout of our Michelson-like interferometer is depicted in Fig 2. In addition to the sample and reference arm, it features a recalibration arm, which is used to automatically prerecord a trace for later-on k-space resampling. This makes k-clocking redundant and is possible because the FDML features a very low sweep-to-sweep variation of the wavelength-over-time dependence. Hence, the same curve is used for all recalibrations. The signals of the sample and reference arm are overlaid in two spectrally equalized and flattened output ports, which are connected to a dual balanced photo detector (BPD). Depending on the setup, the BPD has 1 GHz bandwidth (Wieserlabs WL-BPD1GA) for *non-live imaging* and 1.6 GHz bandwidth (Thorlabs PDB480C-AC) for *live imaging*.

The sample arm is configured similar to the “60° imaging setup” as described in [50]: The beam is collimated with an aspherical lens with 11 mm focal length (Thorlabs C220TMD-C) to avoid spherical aberrations. Then it is reflected from a resonant scanner (EOPC SC-30) for beam steering on the x-axis and a standard galvanometer scanner (Cambridge Technology 6215H) for beam steering on the y-axis. The center between the two scanner pivot points is relay imaged to the nodal point of the eye via a telescope consisting of two groups of four standard plano-convex spherical lenses each lens with 150 mm and with 300 mm focal length, respectively. A dichroic mirror (Layertec 3” mirror with HRu(45°,1000-1090nm)>99.9% coating) is used to couple an attenuated LED projector into the beam path serving as a fixation target.

**Fig 3:**
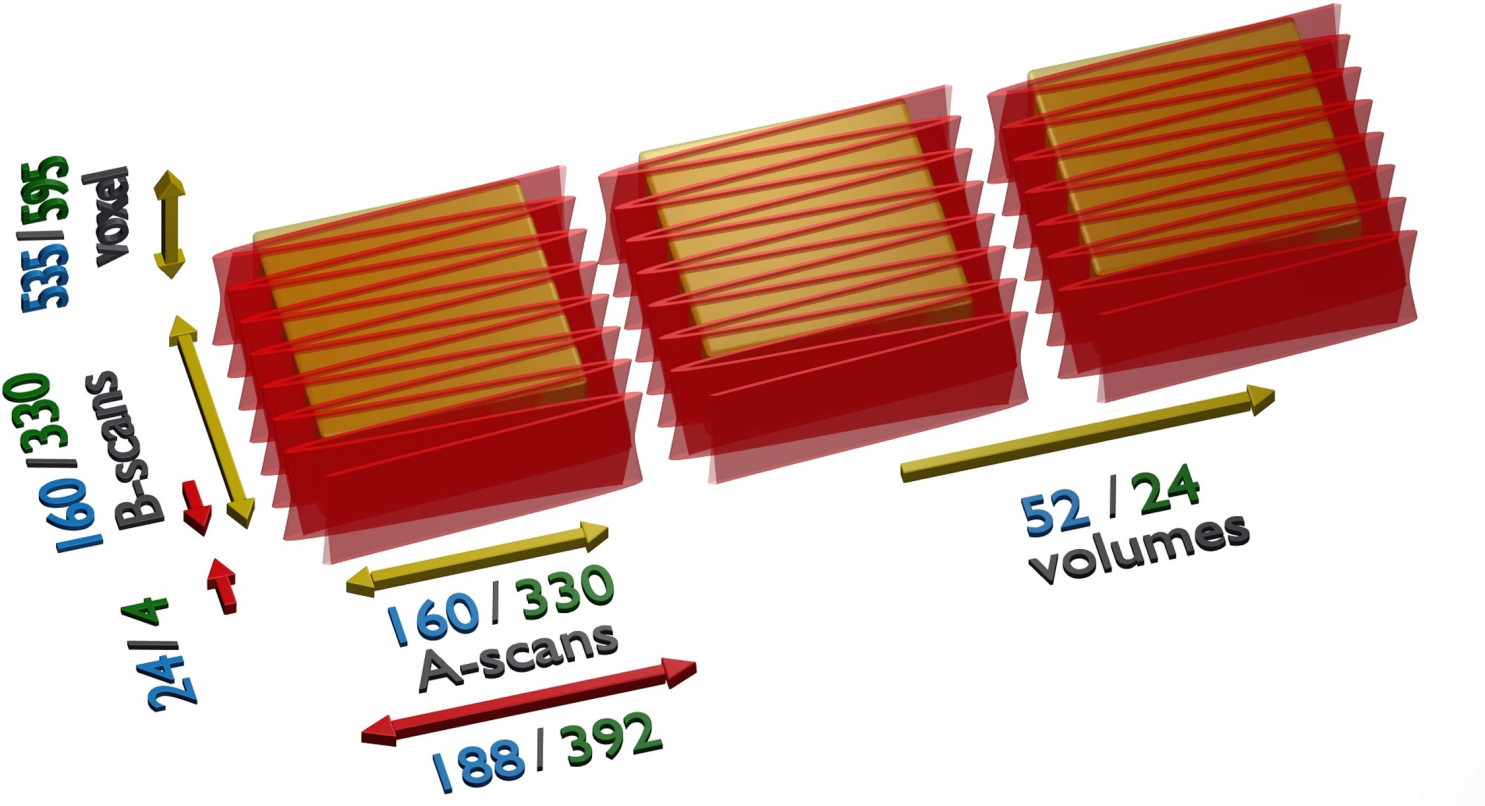
Scan pattern. Red arrows and beam-path denote length of a complete scan. Yellow arrows and box show actual acquired volume. Blue numbers show the non-live imaging case, green numbers the live imaging case.

The resonant scanner has a frequency of 4.294 kHz. We use bidirectional scanning to obtain a frame rate of 8.588 kHz. The FDML frequency corresponds to a 2∙N-multiple of the scanner frequency for synchronization purposes, where N is the buffer factor. Fig 3 explains the resulting scanning protocols. In combination with the 3.34 MHz sweep rate this results in 392 A-scans per B-scan. For the 1.67 MHz we have 188 A-scans per B-scan. As only the most linear 85% of the sinusoidal scan are used, 330 or 160 A-scans remain per B-scan, respectively. Since we aim for an isotropically dense sampling, the same number of B-scans per volume is used. The y-scanner is operated unidirectionally with a few frames (24 for 4x buffering/4 for 8x buffering) for flyback. This gives a volume rate of 52.4 Hz for the 1.67 MHz system and 24.2 Hz for the 3.34 MHz system. In the first case, we reduced the number of B-scans by a factor of two and four to increase the volume rates to 102.2 Hz and 191.2 Hz, respectively. The corresponding fields of view for each configuration are 15°×15°, 15°×7° and 15°×3° for *non-live imaging* and 25°×25° for *live imaging*.

### 2.3 Data acquisition and processing

Digitization was performed with a 1.8 GSamples/s (GS/s) analog to digital converter card (ADC, Alazartech ATS9360) in case of the *non-live* videorate configuration and with a 4 GS/s ADC (Alazartech ATS9373) for *live imaging*. Both digitizer cards feature 12 bit sampling depth. This results in about 1100 or 1200 samples per A-scan and half of it after Fourier transform (FFT), resulting in a total number of 0.6 Gvoxel/s or 1.58 Gvoxel/s, respectively. The corresponding imaging ranges are 4.2 mm and 4.7 mm in air. These are much larger than the actual thickness of the retina and choroid. However, one also has to take into account patient movements and additional space for visualization of instruments. Regarding the long imaging range, we verified that the system has a suitable roll-off performance. We measured the roll-off for both FDML lasers. The point spread function decreased by 6 dB at 3.5mm for the 1.67 MHz version. For the 3.35 MHz version, the same decrease was reached at, 1.7 mm. Both values were negatively affected by electronic timing errors in our system, a problem which will be solved in the future.

#### 2.3.1 Non-live imaging processing

In case of *non-live imaging* the data is directly streamed into the 32 GB RAM of the host computer. We acquired 260 successive volumes for each protocol with a custom Labview program running on Windows. The image processing is as following: We start with subtracting the background, resampling the interference fringes, FFT, dynamic range compression and cropping in a custom Labview program. The resulting B-scan images are then imported into Adobe Lightroom 5, where we apply de-noising filters. Similar results can be achieved with the non-local means plug-in in ImageJ [53]. This is a crucial step, as the noise above surfaces would obstruct the view in the 3D rendering. We use a script in ImageJ to render the 3D view of all 260 volumes and to compose them to a video.

#### 2.3.2 Live-image processing

For *live imaging*, we used the OGOP software (Optores GmbH) running on Linux. Here, the 6 GB/s data stream from the digitizer card is directly transferred on the PCIe-bus to an Nvidia Geforce GTX690 dual GPU board. Each GPU alternatingly processes one B-scan. First, the data is unpacked, which is required because the Alazartech ATS9373 packs two 12bit sample points into three bytes to save streaming bandwidth. Next, a significant amount of processing time is spent on resampling the data to a linear k-space. We can use this substitution for a k-clock as our FDML laser shows a very low sweep to sweep variation of the wavelength-over-time correlation. After apodization, the FFT is performed which takes an equivalent amount of processing time as the linearization and finally a dynamic range compression is applied. The processed data is then directly streamed B-scan-wise to an Nvidia Geforce GTX680 single GPU board, which is responsible for rendering the 3D volumes with forward volume ray integration. Due to this architecture a volume is displayed with a time lag of less than one volume, corresponding to roughly to a maximum value of 40ms at 24.2 Hz volume rate.

### 2.4 Synchronization

**Fig 4:**
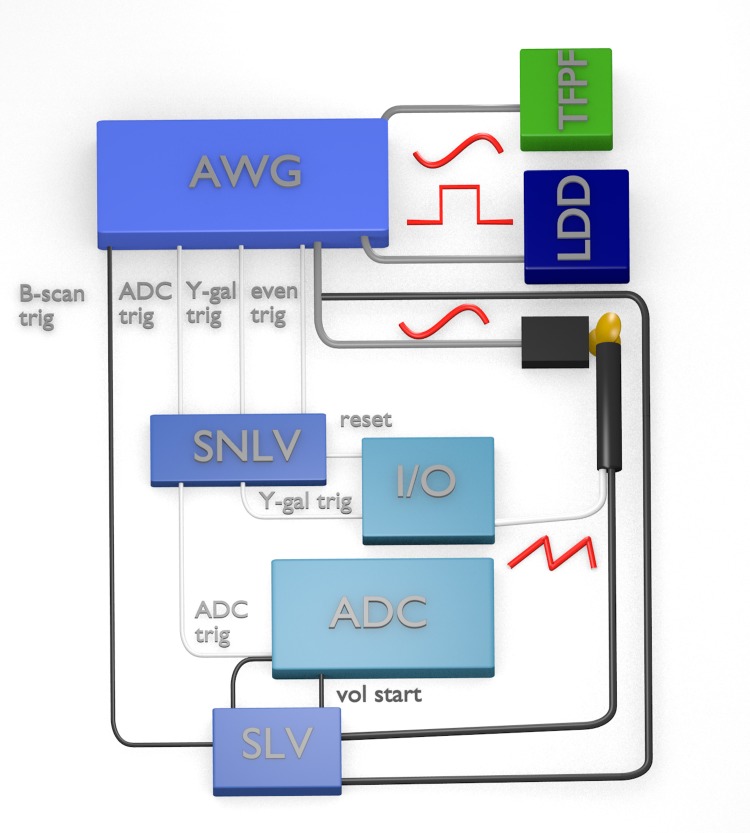
Synchronization of the setup. AWG: arbitrary waveform generator, SNLV: synchronization electronics for non-live imaging, I/O: I/O-card, ADC: data acquisition card, SLV: synchronization electronics for live imaging, TFPF: tunable Fabry-Pérot filter, LDD: laser diode driver. Connections are color and thickness coded: Thick lines transmit analog signals, thin lines transmit digital signals for synchronization. Grey lines are always used, white lines are only used for non-live imaging, and black lines are only used for live imaging. B-scan trig: trigger for B-scans, ADC trig: ADC trigger, Y-gal trig: trigger for y-galvanometer scanner, even trig: even frame trigger. The x-scanner receives a sinusoidal signal, which is also used for even frame synchronization, the y-scanner a triangular signal.

The synchronization scheme of our setup is displayed in Fig 4. We use a slightly different strategy for *live* and *non-live imaging* due to the individual requirements of the two acquisition programs. The difference lies in the control of the Y-scanner and the triggering of the ADC. In each case the central device is a four-channel arbitrary waveform generator (AWG, Thurlby Thandar Instruments TGA12104). It drives the tunable Fabry-Pérot filter (TFPF) with a sinusoidal voltage that is amplified with a home built amplifier. An offset to this voltage is applied via a home built high-voltage bias-T. The second channel of the AWG drives the LDD of the FDML laser with a TTL signal with a duty cycle of 25% or 12.5% for the cases of 1.67 MHz or 3.34 MHz, respectively, for the optical buffering as outlined in subsection 2.1. The third channel gives a sinusoidal clock signal to the driver electronics of the resonant scanner. Each of the three channels is programmed with an arbitrary waveform with the length of two B-scans i.e. one complete cycle of the resonant scanner. The Y-scanner was controlled independently, because the AWG did not provide enough memory to store a volume-long waveform with sufficient resolution.

The AWG also offers a programmable TTL synchronization output (sync out) for each of the four channels. For non-live imaging, three sync outs (ADC trigger, Y-scanner trigger and even frame trigger) are connected to a home-developed synchronization circuit, which ensures that the first B-scan of each volume has the same scan direction. While the ADC and Y-scanner trigger are sent twice per waveform, the even frame trigger occurs only once. After the I/O card (National Instruments PCIe-6351) resets the synchronization circuit via a TTL signal at the beginning of an acquisition, the box starts forwarding the ADC and Y-scanner trigger after one even frame trigger has arrived. The Y-scanner trigger clocks the analog output of the I/O-card, which is connected to the controller of the Y-axis galvanometer scanner. For live imaging, we use a different microcontroller-based synchronization device based on an STM32 (STMicroelectronics Inc.) that also controls the Y-scanner. One sync out of the AWG sends a trigger at the beginning of each B-scan to the device. The I/O port of the ADC is set to high by the OGOP software when it is ready for acquisition. As soon as the device detects a rising edge from the I/O port, it starts scanning the Y-scanner and forwards a B-scan trigger to the ADC card. It stops, when it detects a falling edge. Additionally, the device receives a copy of the resonant scanner signal to ensure correct orientation of the volume.

These strategies have the advantage that the control of the X-scanner by the AWG guarantees a continuous scanning even if the computer freezes. This safety precaution prevents a stationary beam on the retina and makes sure that the optical power is distributed along a line. Nevertheless, we would like to note that even with a stationary beam the exposure of 1.6mW still complies with the American National Standard Institute (ANSI) standards for safe ocular exposure [54].

## 3. 4D MHz-OCT-Imaging

This section presents the imaging results. First, we performed *non-live imaging* with focus on the phase stable application, then *live imaging* was used to evaluate a possible use in surgery. In each subsection, results are presented and discussed focusing on possible challenges. All in vivo retinal imaging experiments were performed in accordance to the tenets of the Declaration of Helsinki. The ethics committee of the University of Lubeck approved the experiments. Verbal informed consent was obtained from the volunteers prior to the measurements. This was in January 2015 for the non-live imaging and in September 2017 for the live imaging. Data of four volunteers (all members of the group) in total was recorded, but not all are presented here for redundancy reasons.

### 3.1 Non-live imaging

**Fig 5:**
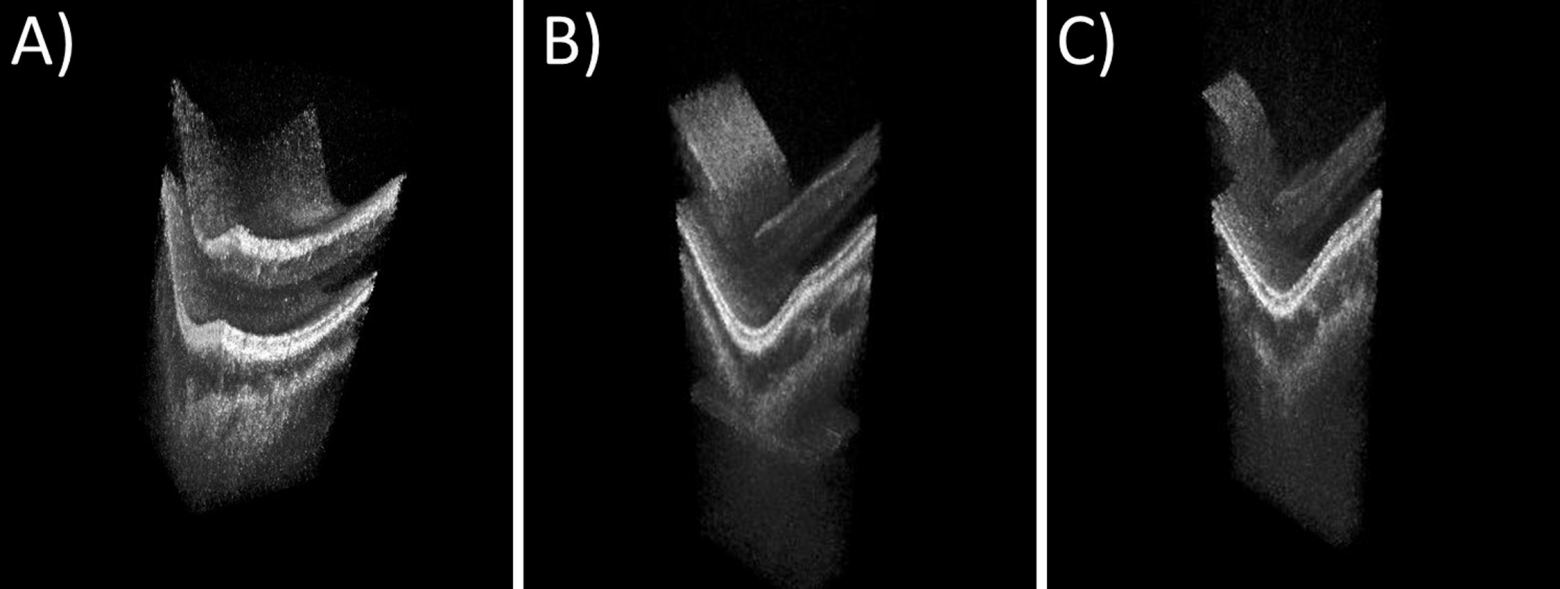
Videos from non-live imaging in real time playback and slow-motion playback with 20 Hz volume rate. A) 160x160 A-scans at 52 Hz with 4.5x4.5x4.2 mm in size(S1 and S2 Videos) B) 160x80 A-scans at 102 Hz with 4.5x2.1x4.2 mm in size (S3 and S4 Videos) and C) 160x40 A-scans at 191 Hz volume rate with 4.5x0.9x4.2 mmin size(S5–S6 Videos).

We measured the sensitivity of our system to be 90 dB which is shot-noise limited [55] if one accounts for losses attributed to the interferometer and the free space optics. A 28 year old healthy male volunteer was imaged with all three scanning protocols in one session. For the two protocols with 52 Hz (A) and 102 Hz (B) volume rate, he was asked to fixate a single target, while he had to fixate multiple targets in sequence for the fast imaging protocol with 191 Hz (C) volume rate. This procedure should simulate very heavy eye motion to evaluate if the system is capable of generating sufficient data quality for phase stable imaging under such circumstances. S1–S6 Videos show the resulting videos. A single frame of each can be found in Fig 5. All videos provide good image quality with penetration down to the sclera. We analyzed the *axial* and *lateral motion* more closely, since they are possible sources of phase distortions. Motion was quantified by manually tracking the lowest point of the fovea throughout the individual volumes.

In cases (A) and (B), no *lateral motion* caused by microsaccades is visible. Literature reports that microsaccades can occur with a mean frequency of down to 0.2 Hz [56]. The two recordings with no intentional motion were up to five seconds long and therefore agree with the literature values. In case (C) with intentional movement of the eye, we observe angular velocities of up to 45°/s, which also seems plausible according to literature [56]. Lateral motion should be no problem with healthy subjects but if this technique will be applied to patients which usually do not have a good fixation, this could turn into a challenge.

*Axial motion* is clearly visible in all cases. We measure ranges of around 25μm with about 250μm/s velocity. Motions occur only occasionally and are not a continuous oscillation. They probably originate from a slight forward and backward movement of the subject’s head. Although we already use a heat-formable mask known from radiation therapy in combination with a chin rest, we believe that there are still improvements possible. The 250 μm/s correspond to a movement of 29 nm per B-scan or a phase shift of less than 1/10 π. As stated in [57] phase errors between - π and π can be corrected. Therefore it should also be possible to correct for this motion between volumes.

### 3.2 Live imaging

**Fig 6:**
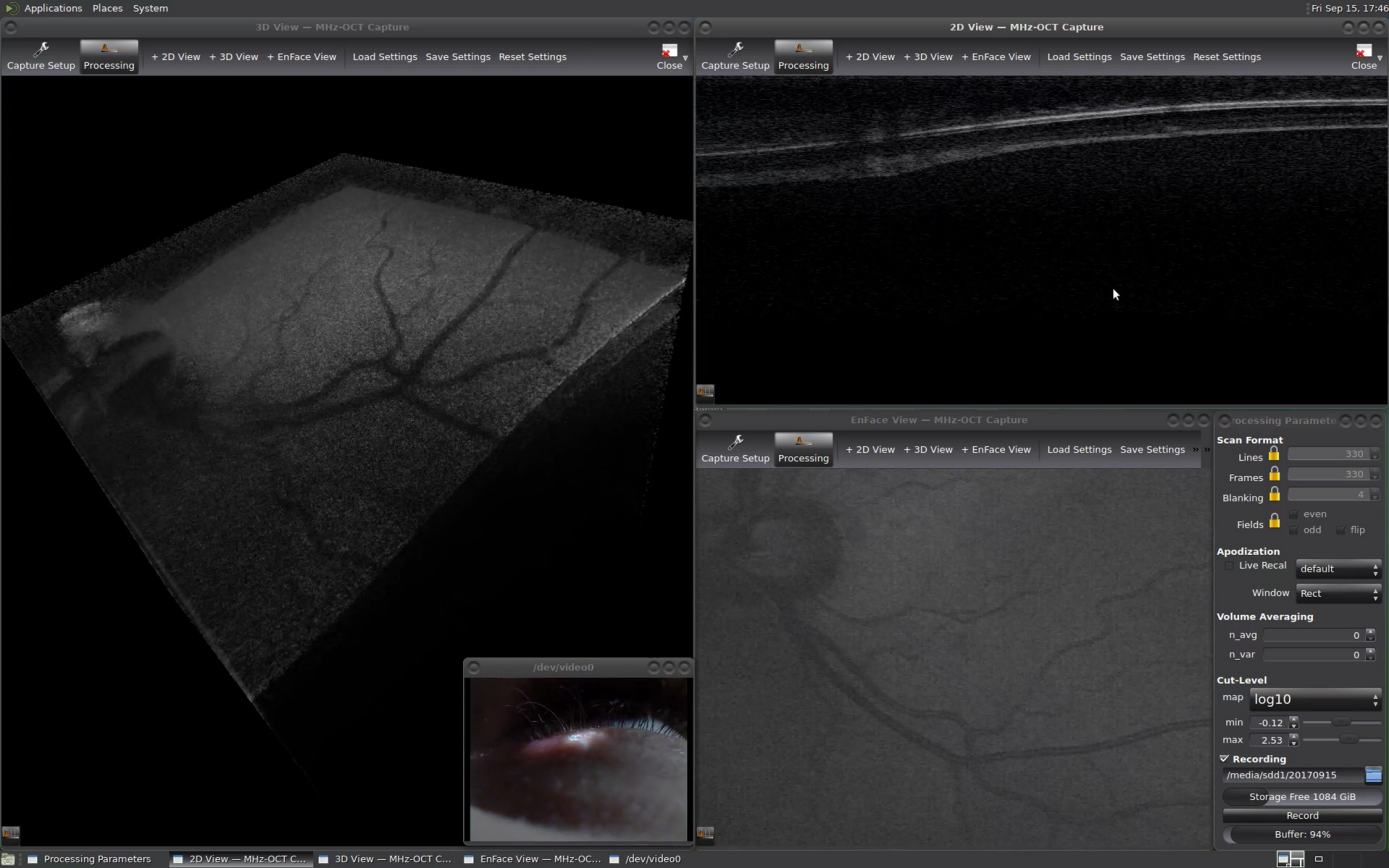
Screen capture recording of live retinal OCT imaging with 330×330×600 voxels at 24.2 Hz volume rate corresponding to 1.58 GVoxel/s (S7 Video). The left window shows the live rendered 3D volume with a size of 7.5x7.5x4.7mm and a webcam stream of the volunteer’s eye in the bottom right corner. On the right side, there is an en face projection (average of the whole volume), a three times averaged B-scan and the program control panel. All images are displayed in a logarithmic scale. A 3D-video in side-by-side format of the imaging session is available in S8 Video.

The system’s sensitivity was measured to be 87dB, this is also shot-noise limited, taking into account the 2x higher speed compared to the previous setup. We imaged a healthy male volunteer at the age of 27. The subject was asked to fixate multiple targets in sequence. Cut levels and transparency were adjusted in the OGOP imaging software until the subjective image quality was optimal. A screen-grabber program (ffmpeg) was used to capture the results of the imaging session and a webcam monitored the volunteer’s eye. The rendered volume was rotated to view it from multiple perspectives. We composed a video showing some exemplary scenes from that (S7 Video), whereas a single frame is shown Fig 6. S8 Video is a side-by-side 3D version of S7 Video.

Main features such as macula, optical nerve head and the shading of blood vessels can be clearly identified in the volumes. The retina shows a high plasticity and all structures are well perceived. If comparing the webcam image and rendered volume in scenes with eye motion, one can appreciate the low time lag in combination with the high volume rate.

However, the choroid and some other layers are not visible in the 3D volume in contrast to the non-live images. One might argue that this is due the lower sensitivity resulting from the higher A-scan rate, but OCT images captured with the same line rate in a previous publication from our group [24] suggest that this is not the case. Compared to the non-live imaging we do not use any denoising filters on the rendered volumes. Therefore, the dynamic range and transparency settings of the volume renderer have to be set such that the noise above the layers is removed completely and only strongly scattering layers remain visible. We believe that more advanced image processing including denoising as demonstrated in [58] should improve the image quality.

**Fig 7:**
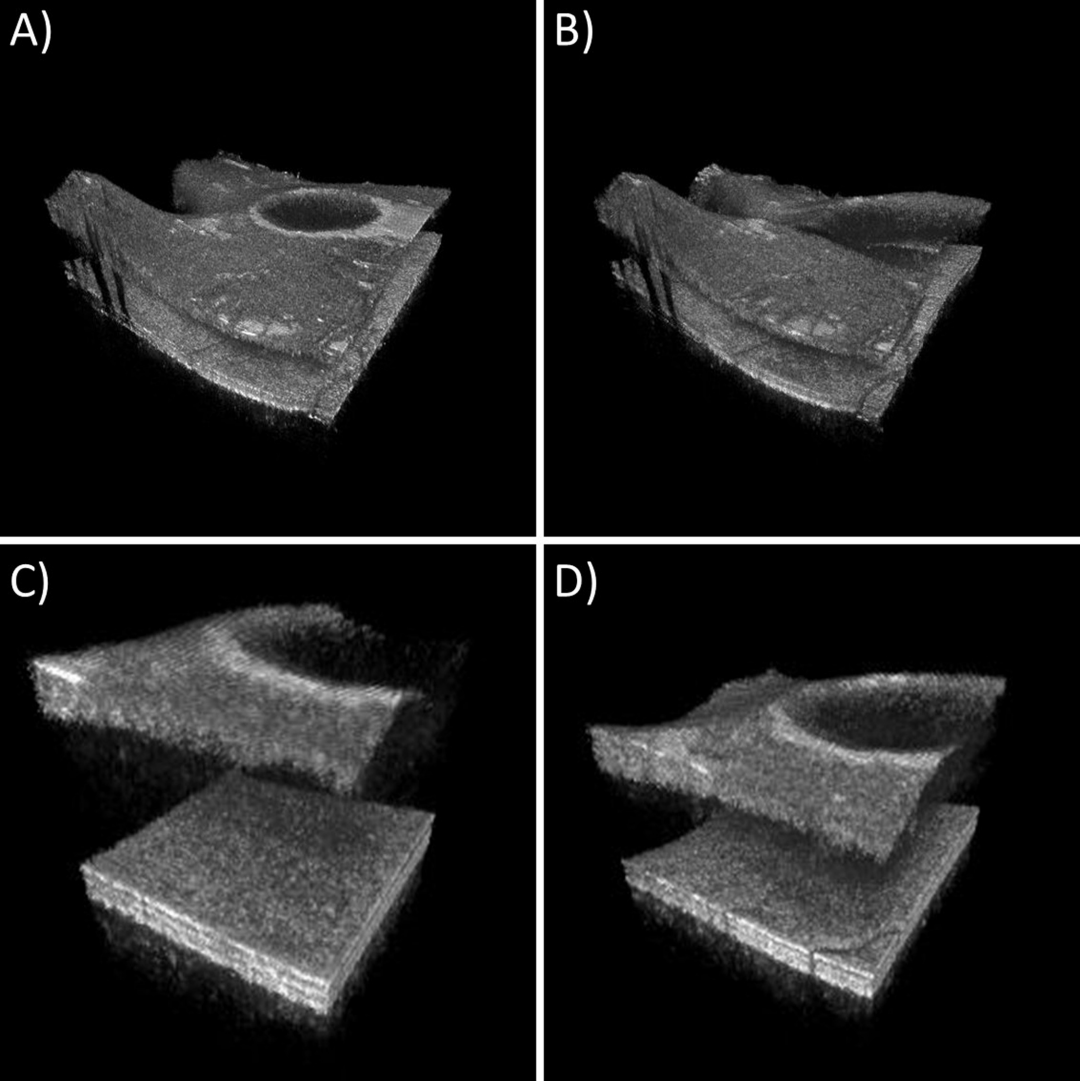
Comparison of original 3.34 MHz A-scan rate recording with simulations of one eighth of the original speed. A) 330×330 A-Scans and 24 V/s original speed (S9 Video). B) 330×330 A-scans and 3 V/s (S10 Video). C) 117×117 A-scans and 24 V/s (S11 Video). D) 165×165 A-scans and 12 V/s (S12 Video).

In order to demonstrate that multi-MHz line rates are required for high-quality live ophthalmic imaging, we recorded raw data during the live imaging, processed it analogous to the non-live imaging and reduced the number of A-Scans by factor of eight. This corresponds to ~400 kHz, which is the fastest possible A-scan rate with current commercially available non-FDML light sources and no spectral splitting [10]. S9–S12 Videos display four different cases (see Fig 7 for frames of the videos): (A) The original speed of 3.34 MHz, (B) a reduction of the volume rate by a factor of eight, (C) a reduction of the volume size by a factor of eight and (D) a reduction of volume rate by a factor of two and volume size by a factor of four. A volume rate of 3 V/s is clearly too slow for a fluid perception of any movements. The 117×117 A-Scans large volume has a far too small field of view. The reduction of both to find a compromise still has a quite small field of view. Here, one might argue, that one could also keep the field of view constant and just reduce the number of A-scans i.e. to perform undersampling. However, this would reduce the transverse resolution and sensitivity. If one would increase the spot size by the same factor, the loss of sensitivity would originate from a reduced collection efficiency–if the spot size was kept constant a washout of the fringes would be responsible [59]. Also the motions appear choppy compared to case (A). Maybe a volume rate of 12 V/s might be sufficient for slow surgical maneuvers, but fast volume rates of 24 V/s or more could be useful for monitoring the tremor of the surgeon’s hand or instrument and other fast surgical dynamics. This suggests clearly that at least 3 MHz are required to have a sufficient field of view and a sufficient smooth motion. The limited sensitivity at such high A-scan rates could be of concern, but we did not use the maximum permissible exposure for safety reasons. Higher optical powers and therefore sensitivities would be possible if safety mechanisms detecting a malfunction of the scanner system would be in place [60].

The current bottleneck of the live processing is the PCI Express 3.0 bus and the ADC. The bus has a theoretical bandwidth limit of 985 MByte/s per lane [61] and the ADC board features 8 lanes resulting in 7.8 GByte/s. The Alazartech ATS9373 has 12 bit sampling depth and 4 GS/s, which result in a data-stream of 6 GByte/s due to packing two samples into three bytes. If one includes some overhead caused by the transfer protocol, there is no extra space for a significantly higher sample rate. Higher sampling rates with streaming would be possible if one would reduce the sampling depth to 8 bit, use a 16-lane interface or in future, the new PCI Express 4.0 bus featuring a doubled transfer rate. However, there are no faster ADCs with streaming capabilities available right now. In order to achieve higher sampling rates with current ADCs, it would be possible to split the signal from the BPD and down-mix the higher frequency components with a local oscillator. The low frequency components are digitized with one ADC corresponding to the first half of the imaging range and the down-mixed high frequency components with another one corresponding to the second half of the frequency range. This scheme is also used in high-speed oscilloscopes. If the digitizer speed cannot be increased it would be also possible to use the available imaging range more efficiently by tuning a local oscillator according to the current image to shift its position [62]. As usually only a small portion of the imaging range is used and most of it is for compensation of motion, one would not loose information.

The available graphics boards are currently no limiting factor to the throughput of our system. We would like to emphasize that the Nvidia GTX680 and GTX690 we use came to the market in 2012. A more recent GPU like the Nvidia GTX 1080 Ti provides a significantly higher processing power and could easily handle higher data rates from the digitizer and a more advanced rendering.

In order to increase not only the imaging range with a higher acquisition rate, but also the volume size and rate, a higher A-scan rate would be required. We demonstrated that higher sweep frequencies are possible with FDML lasers [29]. Alternatively a parallelization approach could be used as we showed in [24]. There is always a tradeoff between speed and sensitivity. Even though we did not use the maximum permissible exposure for a fast scanning system [60], there might be a point where sensitivity becomes too low for a proper visualization Translational studies need to be carried out to find the minimum required sensitivity required for OCT-assisted surgery.

## 4. Conclusion and outlook

This paper investigated the present and possible challenges related to *non-live* and *live* 4D-OCT imaging of the posterior eye with a MHz-FDML laser. We gave detailed information on our setup including the laser source, interferometer, scanning protocols, data acquisition and the necessary synchronization. Videos for both cases were presented and the individual challenges discussed.

We have demonstrated *non-live* retinal imaging with up to 191 V/s, which is unprecedented for flying spot OCT. Our measurements indicate that at this speed motion artifacts should be small enough to enable phase-based image processing to our data, like aberration corrections or pulsation measurements. Larger volumes or even higher sweep rates would be possible with a higher-frequency resonant scanner and the 3.34 MHz FDML laser used in the live imaging.

Additionally, we demonstrated *live* retinal volumetric OCT imaging with 1.58 GVoxel/s which sets a new record for live OCT processing throughput. We could meet all the criteria for an ophthalmic OCT-only surgical microscope stated in the beginning of this article: (1) 330×330×595 voxel large volumes, (2) 24 Hz volume rate, (3) 87 dB sensitivity, (4) a small time lag below 50 ms permitting almost instantaneous feedback to the user, (5) a center wavelength of 1060 nm and (6) a sufficiently large imaging range of 4.7 mm including an appropriate roll off performance. We showed that multi-MHz A-scan rates are necessary for such a microscope. There is also potential for even larger volumes, larger fields of view or higher volume rates, which could be realized by utilizing more or faster digitizers, FDML lasers with higher sweep rates and an up-to-date graphics board. In order to maintain sensitivity, higher exposure powers in combination with scanner failsafe mechanisms would be possible. The image quality was not optimized yet, but we plan to improve it by implementing de-noising and other filters into our live volume renderer [58]. A future step in this project would also be the integration of our approach into a surgical microscope [63] together with simulating surgical maneuvers on phantoms in a larger scale study and a more advanced visualization of the 3D volume [64]. It should also be possible to implement a multi-laser scanning laser ophthalmoscope to map actual colors onto the OCT data to provide even more information to the surgeon. With these improvements in place, a clinical trial of such a microscope would be the next step to show the benefits of future OCT-assisted surgery and to assess necessary parameters in more detail such as the required sensitivity.

## Supporting information

S1 Video Volumetric video rate OCT data with 160x160 A-scans at 52 Hz in real time playback. (MP4)

S2 Video Volumetric video rate OCT data with 160x160 A-scans at 52 Hz in slow-motion playback. (MP4)

S3 Video Volumetric video rate OCT data with 160x80 A-scans at 102 Hz in real time playback. (MP4)

S4 Video Volumetric video rate OCT data with 160x80 A-scans at 102 Hz in slow-motion playback. (MP4)

S5 Video Volumetric video rate OCT data with 160x40 A-scans at 191 Hz in real time playback. (MP4)

S6 Video Volumetric video rate OCT data with 160x40 A-scans at 191 Hz in slow-motion playback. (MP4)

S7 Video Screen capture recording of live retinal OCT imaging with 330×330×600 voxels at 24.2 Hz volume rate. (MP4)

S8 Video A side-by-side 3D-video of S7. (MP4)

S9 Video Volumetric video rate OCT data with 330x330 A-scans at 24 Hz (original imaging speed). (AVI)

S10 Video Volumetric video rate OCT data with 330x330 A-scans at 3 Hz (one eighth imaging speed). (AVI)

S11 Video Volumetric video rate OCT data with 117x117 A-scans at 24 Hz (one eighth imaging speed). (AVI)

S12 Video Volumetric video rate OCT data with 165x165 A-scans at 12 Hz (one eighth imaging speed). (AVI)
