# Correction: Live video rate volumetric OCT imaging of the retina with multi-MHz A-scan rates

**DOI:** 10.1371/journal.pone.0220829

**Published:** 2019-07-31

**Authors:** Jan Philip Kolb, Wolfgang Draxinger, Julian Klee, Tom Pfeiffer, Matthias Eibl, Thomas Klein, Wolfgang Wieser, Robert Huber

Fig 2 is incorrect. The authors have provided a corrected version here.

**Fig 2:**
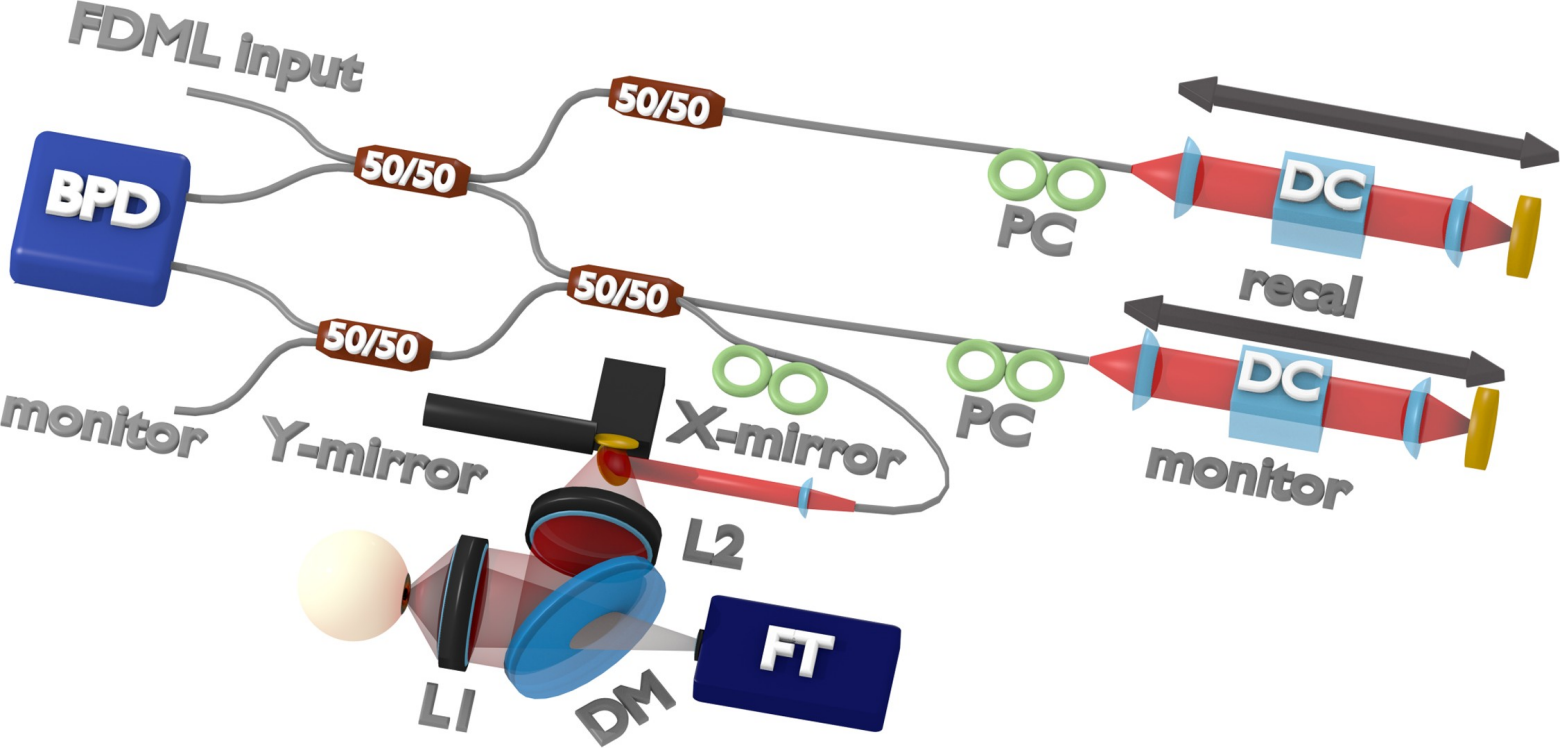
Interferometer design including sample arm. DC: dispersion compensation. BPD: balanced photodetector, recal: recalibration arm, reference: reference arm, PC: polarization controller, X-mirror: x-axis resonant scanner, Y-mirror: Y-axis galvanometer scanner, L1 and L2: lens group 1 and 2, DM: dichroic mirror, FT: fixation target (attenuated LED projector).
